# Survival in Southern European patients waitlisted for kidney transplant after graft failure: A competing risk analysis

**DOI:** 10.1371/journal.pone.0193091

**Published:** 2018-03-07

**Authors:** Domingo Hernández, Alfonso Muriel, Pablo Castro de la Nuez, Juana Alonso-Titos, Pedro Ruiz-Esteban, Ana Duarte, Miguel Gonzalez-Molina, Eulalia Palma, Manuel Alonso, Armando Torres

**Affiliations:** 1 Nephrology Department, Carlos Haya Regional University Hospital and University of Malaga, IBIMA, REDinREN (RD16/0009/0006), Malaga, Spain; 2 Clinic Biostatistic Unit, Hospital Ramón y Cajal IRYCIS, CIBERESP, Universidad Autónoma, Madrid, Spain; 3 Transplant Coordination Center and Andalusian Health Service, Seville, Spain; 4 Nephrology Department, Hospital Universitario de Canarias, CIBICAN, University of La Laguna, REDinREN (RD16/0009/0031) and Instituto Reina Sofía de Investigación Renal (IRSIN), Tenerife, Spain; Icahn School of Medicine at Mount Sinai, UNITED STATES

## Abstract

**Background:**

Whether patients waitlisted for a second transplant after failure of a previous kidney graft have higher mortality than transplant-näive waitlisted patients is uncertain.

**Methods:**

We assessed the relationship between a failed transplant and mortality in 3851 adult KT candidates, listed between 1984–2012, using a competing risk analysis in the total population and in a propensity score-matched cohort. Mortality was also modeled by inverse probability weighting (IPTW) competing risk regression.

**Results:**

At waitlist entry 225 (5.8%) patients had experienced transplant failure. All-cause mortality was higher in the post-graft failure group (16% vs. 11%; *P* = 0.033). Most deaths occurred within three years after listing. Cardiovascular disease was the leading cause of death (25.3%), followed by infections (19.3%). Multivariate competing risk regression showed that prior transplant failure was associated with a 1.5-fold increased risk of mortality (95% confidence interval [CI], 1.01–2.2). After propensity score matching (1:5), the competing risk regression model revealed a subhazard ratio (SHR) of 1.6 (95% CI, 1.01–2.5). A similar mortality risk was observed after the IPTW analysis (SHR, 1.7; 95% CI, 1.1–2.6).

**Conclusions:**

Previous transplant failure is associated with increased mortality among KT candidates after relisting. This information is important in daily clinical practice when assessing relisted patients for a retransplant.

## Introduction

Loss of a kidney graft has emerged as an important cause of end-stage renal disease (ESRD). Most affected patients need to return to dialysis, with some relisted for transplantation [1,2].

Patients on the waiting list for kidney transplantation (KT) have a lower mortality risk than those not listed [3]. However, patients relisted after graft failure have a decreased survival compared with transplant-naïve (TN) patients waitlisted for KT [4–7]. Transplantation is not usually considered a classical risk factor for death, but KT is generally associated with a high burden of immunosuppression and comorbid conditions, which could lead to life-threatening complications and thus increase mortality when these patients are relisted. Additionally, whether the effect of a prior KT on survival of relisted patients changes over the time these patients are on the waiting list is undetermined. The question, therefore, is whether patients who are waitlisted following a failed KT are at increased risk for death relative to TN patients waitlisted for KT. Accordingly, a more appropriate comparison group for patients waitlisted after graft failure would be patients deemed eligible to receive a primary transplant.

Observational studies have assessed survival of patients returning to dialysis after graft failure, but most studies involved heterogeneous patient groups (e.g. waitlisted and not waitlisted) or were single-center studies with relatively small sample sizes and of limited generalizability [4–13]. Consequently, explicit comparisons of mortality between patients waitlisted after graft failure and TN patients waitlisted for KT are lacking [5], especially studies that use proper assessment of survival in this particular population, such as a competing risk analysis, where transplantation may be considered a competing risk when evaluating survival in waitlisted patients [14,15]. In addition, as transplant clinicians do not randomly allocate waitlisted patients for KT a propensity score analysis should be performed when assessing survival to avoid potential selection bias between waitlisted patients who have received a prior transplant and TN waitlisted patients [16]. Importantly however, no large-scale study has yet used a competing risk approach and propensity score analyses to address the question of whether patients who are relisted following a failed transplant are at increased risk for death relative to waitlisted patients who have not yet received a transplant. In light of this, therefore, we undertook competing risk analyses and a propensity score analysis to compare survival of patients waitlisted for KT after graft failure with that of TN waitlisted patients. We hypothesized that relisted patients have an increased risk of mortality.

## Material and methods

### Study population

**Fig 1:**
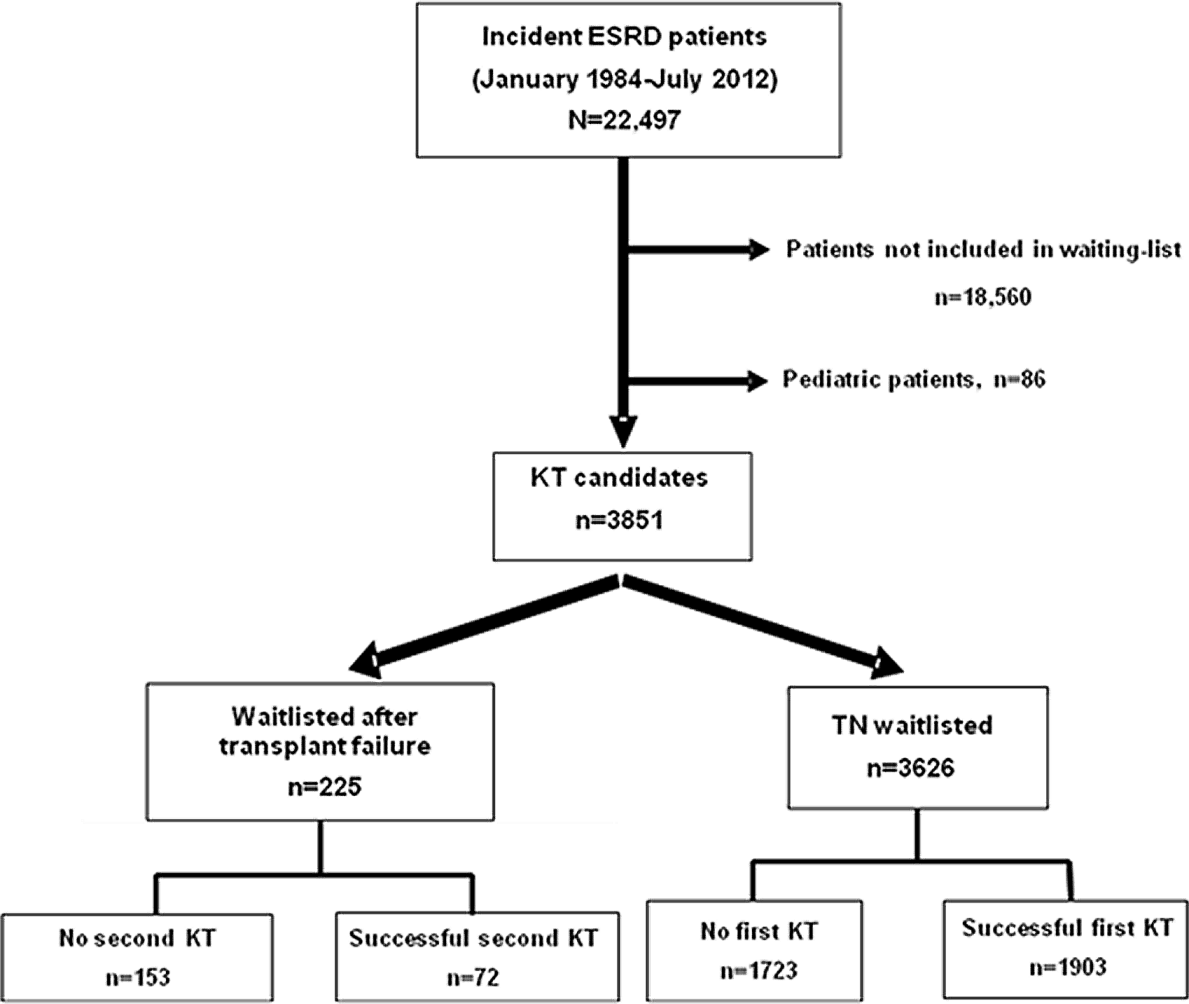
Flow chart of the study population. ESRD, end-stage renal disease; KT, kidney transplant; TN transplant-näive.

This longitudinal cohort study involved 22,497 renal patients included in the Andalusian Registry of Renal Patients between January 1, 1984, and July 31, 2012, because of initiation of dialysis. Data from all centers in Andalusia, a region in southern Spain with nearly 9 million inhabitants, were entered into the registry with follow-up information through July 31, 2012. The database is updated annually and the degree of compliance of data concerning waitlist patients was almost universal. Details of the study design have been reported, as have the baseline clinical and demographic data of the study population [14]. Briefly, we excluded 18,560 patients not listed for KT at entry to dialysis, including pre-emptive KT, and 86 patients younger than 18 years. We therefore assessed a final total population of 3851 adult KT candidates (225 relisted after a failed graft and 3626 TN). Of these, 1975 received a KT (72 a retransplant and 1903 TN) and 1876 were on the waitlist at any time during the study period (153 relisted and 1723 TN) (Fig 1). Of the 72 retransplanted patients, 5 died, 2 experienced graft loss due to chronic allograft nephropathy and one was lost to follow-up. The overall total median time on the waiting list was 21.2 months (interquartile range, 11–37.4). The clinical characteristics of the waitlisted patients and the KT recipients are shown in supplementary material (S1 Table). A higher age and a greater proportion of comorbidities were observed in the waiting-list group compared with the patients who received a KT [14]. Inactive status was defined as candidates not suitable for transplantation, for whatever reason, at the time of waitlist inclusion or while on the waiting list (incomplete workup, medical noncompliance, inappropriate weight, temporarily too sick, etc.), but still an appropriate patient to remain on the waiting list. Consequently, we also took into account “inactive status” within the waitlist (n = 316) when performing survival analyses because these patients had not been definitively removed from the waitlist. We therefore evaluated survival of all patients who remained on the waitlist during follow-up, including inactive status patients.

Some recommendations for keeping a patient on the waiting list or removing someone from it are broad rather than specific and are based on clinical practice guidelines on waitlisting for KT [17,18].

The following data were recorded at the start of dialysis: (a) demographic and clinical data: age, sex, cause of ESRD and comorbidities compiled in the Charlson comorbidity index (CCI) [19]: myocardial infarction and congestive heart failure (grouped as cardiac disease), peripheral vascular disease, hemiplegia, diabetes, connective tissue disease, mild liver disease (considered as the presence of hepatitis B surface antigen or positive hepatitis C virus antibodies without cirrhosis), cirrhosis, chronic pulmonary disease, peptic ulcer, any tumor without metastasis, and HIV infection; (b) conditions and comorbidities inherent to the uremia: dialysis modality at entry (hemodialysis versus peritoneal dialysis), presence of a central venous catheter (CVC) at dialysis entry regardless of dialysis modality, time on waitlist (defined as time from waitlist inclusion until end of follow-up or permanent or temporary withdrawal from the waiting list), total time on dialysis (defined as time from starting dialysis until end of follow-up, including time on waitlist for a first KT and time on dialysis whist awaiting a second, third or fourth transplant–that is, the sum of all periods on dialysis throughout the follow-up), and previous transplant, considering also as a covariate one or more previous transplants; and (c) community risk factors, e.g. early or late referral to the nephrologist (considered as a cut-off time of 6 months) and employment status (employed vs. unemployed or retired because of age or disability). Lastly, the waitlisted year was also registered, grouped in 4- or 5-year periods (1984–1987 vs. 1988–1992 vs. 1993–1997 vs. 1998–2002 vs. 2003–2007 vs. 2008–2012).

Medical record review was approved by the ethics committee of Carlos Haya Regional University Hospital and conducted according to the Declaration of Helsinki. As the patient data in the third-party database were de-identified prior to access by the authors, the ethics committee waived patient approval. The datasets can be accessed in the same manner as the authors via a request to the Transplant Coordinator of the Andalusian Health Service (Seville, Spain). The authors themselves had no special access privileges.

### Outcome

Mortality whilst on the waitlist was the clinical endpoint. Survival was measured in months from waitlist inclusion, with patients censored at the time of permanent or temporary waitlist withdrawal for any reason, including KT, inactive status or last follow-up (31 December, 2012). We took into account competing events. Thus, survival analyses were performed using a competing risk approach, where KT and inactive status condition were treated as competing events. Survival data were available for the whole population and all deaths while on the waiting list were recorded. Survival analysis considered the whole study population; that is, all active and inactive status patients on the waitlist. Survival was also analyzed excluding inactive status and diabetic patients.

### Statistical analysis

Continuous data were expressed as mean±standard deviation or median and interquartile range. Comparisons of continuous variables between patients waitlisted after graft failure and TN patients waitlisted for KT were performed using unpaired *t-*test. Absolute and relative frequencies were used to describe categorical variables, which were compared between both groups using the chi-square test.

As a result of an imbalance in clinical characteristics between patients waitlisted after a prior transplant and TN patients waitlisted for KT, a propensity score-matched pair analysis was used to compare outcomes between the two patient groups with similar predicted probabilities of receiving a previous KT. We therefore created a multivariable logistic regression model to estimate the propensity score for receiving a previous transplant by using a greedy matched pair analysis that has a 1:5 matching algorithm with replacement [20,21]. Covariates used for this analysis were the clinical characteristics of the patients, including comorbidities, diabetes condition, time on dialysis, listing year grouped in 4- or 5-year periods (1984–1987 vs. 1988–1992 vs. 1993–1997 vs. 1998–2002 vs. 2003–2007 vs. 2008–2012), a CVC, late nephrologist referral, employment status and dialysis modality. To assess the effectiveness of bias reduction after the matching procedure we measured absolute standardized differences, expressed as a percentage of the pooled standard deviation, and compared differences between matched groups [22]. We evaluated iteratively various caliper widths until between-group standardized differences were minimized. The final selected caliper width was 0.05 and TN waitlisted patients were sampled with replacement. As a sensitivity analysis we also explored a different method, inverse probability weighting (IPTW), to assess the issue of confounding for indication [23].

We used a competing risk approach where KT and inactive status condition were managed as competing events that compete with our clinical end-point. In particular, competing risk regression models were applied directly to the cumulative incidence function for particular use in competing risks analyses, as described by Fine and Gray [24]. Three strategies were used to assess the association between prior KT and mortality in waitlisted patients by using competing risk regression models. Firstly, we used a multivariate adjustment competing risk model in the total study population. Adjustment covariates included comorbidities compiled in the CCI, including diabetes condition, age, sex, presence of a CVC regardless of dialysis modality, late referral to nephrologist, employment status, dialysis modality, time on dialysis, previous transplant (yes/no), number of previous transplants and listing time periods (1984–87 vs. 1988–92 vs. 1993–97 vs. 1998–2002 vs. 2003–07 vs. 2008–12). Second, a competing risk regression model was fitted to the propensity score-matched cohort. The adjusted model on the matched set included covariates that had standardized differences of >10% [25]. Finally, the primary endpoint was modeled by a IPTW competing risk regression model. These models were fitted using the stcrreg Stata command.

Time on waitlist for both TN waitlisted patients and patients waitlisted after graft failure was defined in our survival analyses as the time from placement on the waiting list (time zero) until permanent or temporary withdrawal for any reason (death, KT or inactive status) or last follow-up (31 December, 2012). The start of the time on the waitlist (time zero) for the post-graft failure dialysis group was defined as the date they were included on the waiting list after returning to long-term dialysis.

We used psmatch2 for the propensity score analyses and SPSS version 15.0 (SPSS, Inc., Chicago, IL) and Stata version 13.1 (StataCorp LP., College Station, TX) for all other analyses. A *P* value <0.05 was considered significant.

### Missing values

Although the data on mortality were complete, some clinical data were not available for all the 3851 patients included in the study. Nevertheless, there were relatively few missing values, including in the subset of patients relisted after graft failure (<10%). Missing data were treated by means of multiple imputation [26]. Because a large subset of the population (93%) had no missing data, final survival analysis was not affected by missing data.

## Results

Of the 3851 patients included in the study, 225 (5.8%) had had a prior transplant at the start of dialysis. Of these 225, 204 had received just one previous transplant, 20 had received two previous transplants and only one had received three transplants. Table 1 summarizes the baseline clinical and demographic characteristics for the patients waitlisted after allograft failure and the TN waitlisted patients. Significant differences were observed between the groups for age, sex, primary cause of ESRD, type of dialysis at entry, late referral to nephrologist and comorbidities, including peripheral vascular disease, mild liver disease and chronic pulmonary disease. In addition, a higher proportion of TN waitlisted patients received a KT (52%), whereas only 32% of patients waitlisted after graft failure received a KT. Accordingly, these patients who had already experienced graft failure had a higher median time on dialysis.

**Table 1:** Clinical and demographic characteristics of patients waitlisted after allograft failure versus transplant-naïve waitlisted patients.

	Patients waitlisted after graft failure (n = 225)	Transplant-naïve waitlisted patients (n = 3626)	*P* value
Mean age, *y*	48.4±14	53.3±13.5	<0.001
Male, *%*	56	63	0.027
Cardiac disease*, *%*	5.3	7.8	0.180
*Heart failure*	4.4	6.5	
*Myocardial infarction*	0.9	1.7	
Hemiplegia	0.9	2.2	0.328
Chronic pulmonary disease, *%*	2.2	5.3	0.041
Connective tissue disorder, *%*	4	4.2	0.905
Primary cause of renal disease *%*			
*Diabetes*	8	17.4	<0.001
*Gomerulonephritis*	21	12	
*Polycystic disease*	11.6	12	
*Nephrosclerosis*	4.4	10	
*Interstitial nephritis*	15	11	
*Unknown*	21	28	
*Other*	18	10	
Mild liver disease^, *%*	8	3	<0.001
Cirrhosis, *%*	0.9	1.2	1.000
HIV positive, *%*	0.4	0.8	1.000
Peptic ulcer, *%*	2.7	2.8	1.000
Any tumor without metastasis, *%*	2.2	3.1	0.687
Hemodialysis at entry, %	73	81	0.005
Central venous catheter, %	29	35	0.071
Peripheral vascular disease, %	4.4	8.2	0.042
Late referral**, *%*	18.7	25.2	0.026
Unemployed status^^, *%*	56	58	0.677
Median CCI (IR)	3 (2–4)	4 (2–5)	<0.001
Median total time on dialysis^+^ (IR), *mo*	24 (11–55)	21 (11–38)	0.037
KT recipients, *%*	32	52	<0.001

*Cardiac disease was considered as heart failure or myocardial infarction.

^Mild liver disease was considered as the presence of hepatitis B surface antigen or positive hepatitis C virus antibodies without cirrhosis.

** A 6-month cut-off time was defined for late referral to nephrologist

^^Unemployed status was considered as unemployed or retired because of age or disability

^+^Total time on dialysis was defined as time from starting dialysis until end of follow-up, including time on waitlist for a first KT and time on dialysis whilst awaiting a second, third or fourth transplant. Thus, total time on dialysis represents the sum of all periods on dialysis throughout the follow-up.

Abbreviations: PVD, peripheral vascular disease; HIV, human immunodeficiency virus; CCI, Charlson comorbidity index; KT, kidney transplantation; IR, interquartile range.

After 1:5 propensity score matching, differences across the two groups diminished substantially (Table 2), reflected by a reduction in the absolute standardized difference across almost all the clinical data analyzed when assessing matched cohorts.

**Table 2:** Clinical characteristic of patients waitlisted after graft failure versus transplant-näive waitlisted patients after matching 1:5 with replacement.

	Patients waitlisted after graft failure (n = 225)	Transplant-naïve waitlisted patients (n = 667)	Absolute standardized difference
Mean age, *y*	48.4±14	46.7±14	11.6
Cardiac disease*, *%*	5.3	6.1	2.9
Hemiplegia, *%*	0.9	1.1	2.2
Chronic pulmonary disease, *%*	2.2	2.1	0.5
Connective tissue disorder, *%*	4	4.5	2.7
Diabetes, *%*	8	8.3	1.1
Mild liver disease^, *%*	8	5.6	10.5
Cirrhosis, *%*	0.9	0.5	3.5
HIV positive, *%*	0.4	0.7	3.4
Peptic ulcer, *%*	2.7	1.3	8.1
Any tumor without metastasis, *%*	2.2	2	1.1
Hemodialysis at entry, *%*	73	73.5	1.5
Central venous catheter, *%*	29	25	7.8
Peripheral vascular disease, *%*	4.4	4	1.8
Late referral**, *%*	18.7	20.8	5.2
Unemployed status^^, *%*	56	55	2.9
Listing period, *%*			
*1988–1992*	11.1	10.3	3.5
*1993–1997*	12.4	12	2.2
*1998–2002*	10.6	12.4	7.4
*2003–2007*	41.3	40.8	1.1
*2008–2012*	16.4	16.3	0.2

*Cardiac disease was considered as heart failure or myocardial infarction.

^Mild liver disease was considered as the presence of hepatitis B surface antigen or positive hepatitis C virus antibodies without cirrhosis.

** A 6-month cut-off time was defined for late referral to nephrologist

^^Unemployed status was considered as unemployed or retired because of age or disability

Abbreviations: HIV, human immunodeficiency virus.

### Survival

**Fig 2:**
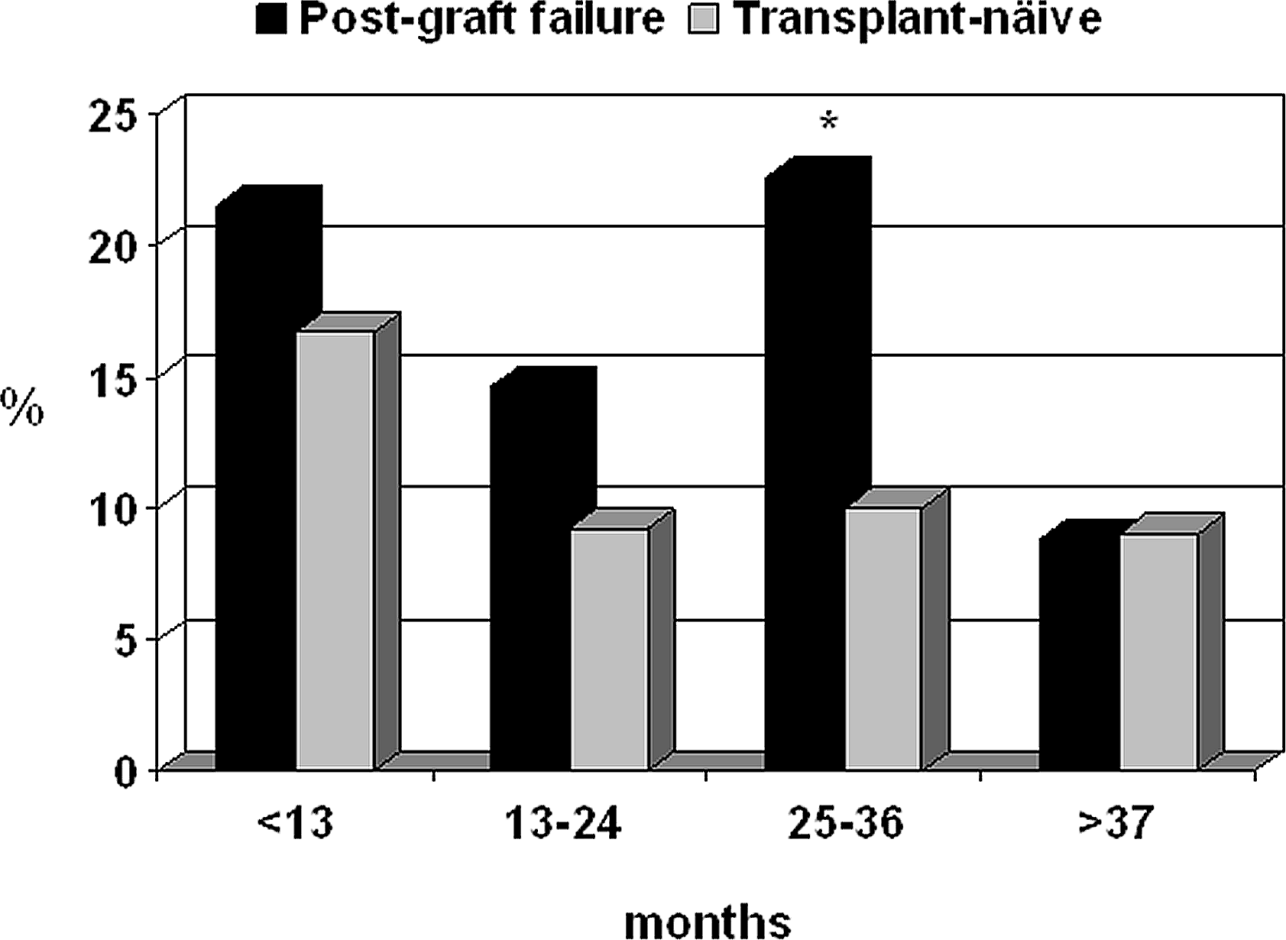
Proportion of deaths in both groups of waiting-list patients according to time on list. Overall comparison: chi-square = 42.8; *P*<0.0001 *P = 0.029 vs. transplant-näive waitlisted patients (chi-square = 4.77).

**Fig 3:**
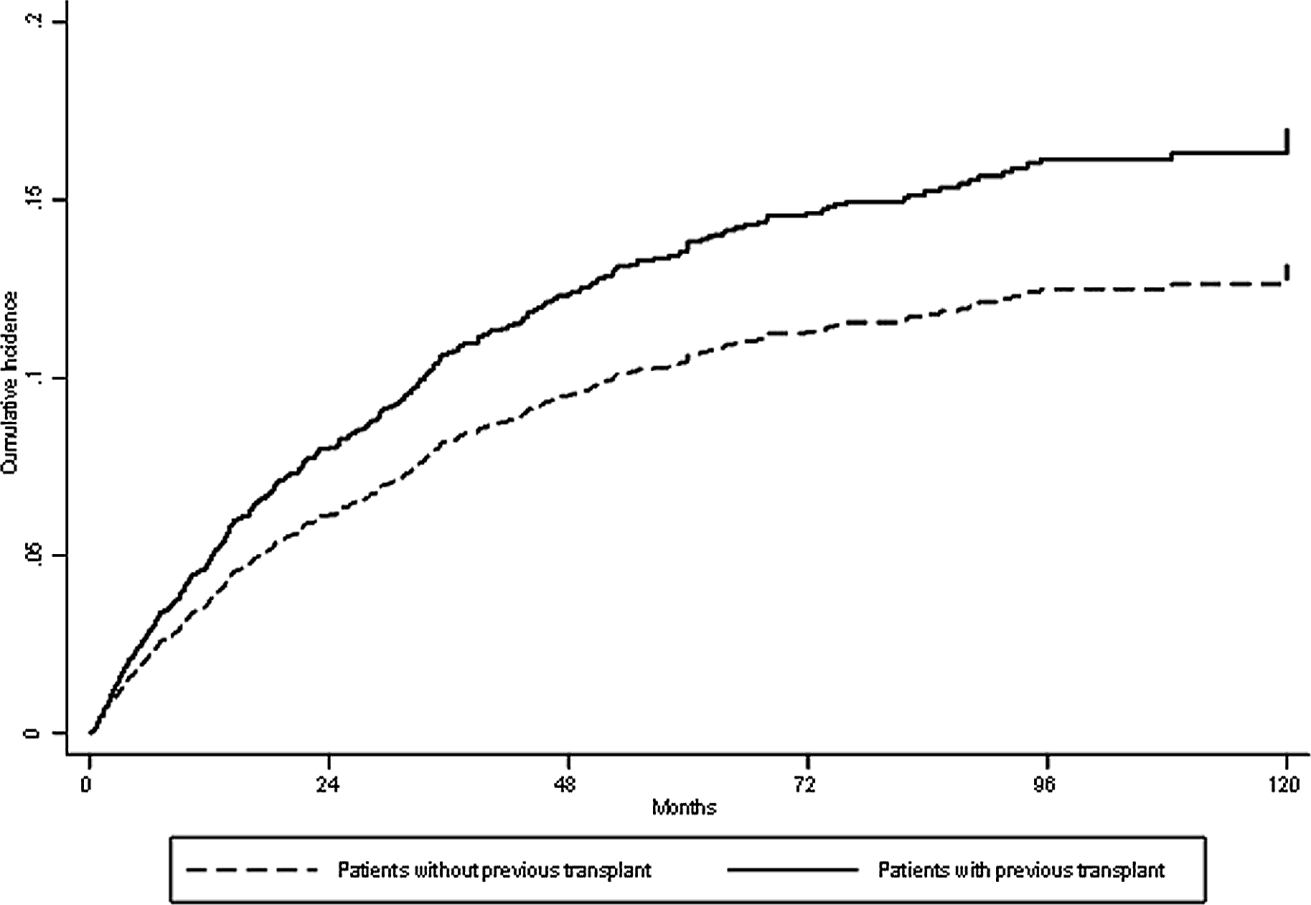
Estimated survival curves for death while on the waiting list in both groups adjusted for confounders using competing risk analysis.

The overall median follow-up was 22 months (interquartile range 11–39 months). A total of 446 (24%) patients died while they remained on the waiting list (n = 1876) (Table 3). Overall, cardiovascular diseases were the leading cause of mortality (25.3%), followed by infectious complications (19.3%), cancer (7.2%) and hepatic disorders (3%). Miscellaneous causes, including those from an unknown source, accounted for 46%. Of note, patients waitlisted after graft failure experienced a higher all-cause mortality than waitlisted TN patients (16% vs. 11%; *P* = 0.033). Likewise, a trend toward higher cardiovascular mortality was seen in patients waitlisted after graft failure compared with waitlisted TN patients (Table 3) despite the former being younger and having a lower burden of comorbidities at dialysis entry (Table 1). Most deaths of waitlisted patients occurred within the first three years after listing and a significantly higher mortality occurred among those patients waitlisted after graft failure versus waitlisted TN patients (chi-square 42.8; *P*<0.0001), especially at the third year post-listing (Fig 2). Fig 3 shows the cumulative incidence of deaths while on the waiting list over time in both groups according to the propensity score-matched analysis. Finally, among the patients waitlisted after graft failure who died (n = 36), 33 had received only one previous transplant, while three had received two transplants.

**Table 3:** Causes of death among patients who remained on the waiting list^a^ (n = 1876 patients and 446 deaths) after allograft failure versus transplant-naïve waitlisted patients.

Cause of death	Patients waitlisted after graft failure (n = 36)	Transplant-naïve waitlisted patients (n = 410)
Cardiovascular^b^	11 (30.6)	101 (24.6)
Infection	8 (22.2)	78 (19)
Neoplasm	2 (5.6)	30 (7.3)
Liver disorder	1 (2.8)	9 (2.2)
Uncertain	10 (27.8)	119 (29)
Miscellaneous	4 (11)	73 (17.8)

^a^Expressed as number (%)

^b^Causes of death from cardiovascular disease included myocardial infarction, heart failure, stroke, arrhythmia, peripheral vascular event, and sudden death

Table 4 shows the results of the competing risk regression model. After adjustment for age, comorbidities included in the CCI, uremia-related factors, several community risk factors, and listing periods, the subhazard ratio (SHR) estimate for mortality in patients waitlisted after graft failure was 1.5 (95% confidence interval [CI], 1.01–2.2). Other risk factors for mortality in the multivariate model were age (SHR 1.04; 95% CI, 1.03–1.05), cardiac disease (SHR 1.7; 95% CI, 1.5–2.2), diabetes (SHR 2.2; 95% CI, 1.7–2.7), connective tissue disorder (SHR 1.7; 95% CI, 1.2–2.6), peripheral vascular disease (SHR 1.8; 95% CI, 1.4–2.3), liver disease (SHR 2.3; 95% CI, 1.5–3.3), the presence of a central venous catheter (SHR 1.8; 95% CI, 1.5–2.2), unemployed status (SHR 1.7; 95% CI, 1.4–2.1 and any tumor without metastasis (SHR 1.7; 95% CI, 1.1–2.6). Estimated survival curves for death while on the waiting list in both groups adjusted for confounders using competing risk analysis are plotted in Fig 3. When a subanalysis was performed excluding those patients who were relisted between 1984–1988 and were under azathioprine-based immunosuppression, the adjusted competing risk regression model revealed a subhazard ratio estimate for mortality of 1.42 (95% CI, 0.94–2.1; *P* = 0.09). The SHR estimate from the propensity score-matched cohort for death was 1.6 (95% CI, 1.01–2.5). Finally, a similar SHR estimate for mortality was observed when the primary endpoint (death) was modeled by IPTW competing risk model (SHR, 1.7; 95% CI, 1.1–2.6) (Table 4).

**Table 4:** Multivariate competing risk analysis for the relationship between post-graft failure and mortality in relisted patients for kidney transplantation^a^.

Statistical models	Competing risk SHR^b^ (95% CI)	*P* value
Adjusted^c^	1.5 (1.01–2.2)	0.045
Propensity score-matched cohort^d^	1.6 (1.01–2.5)	0.042
IPTW^e^ (weighted estimates)	1.7 (1.1–2.6)	0.018

^a^Transplant-näive waitlisted patients is the reference group.

^b^Subhazard ratios are from competing risk models (n = 3851 and 1975 kidney transplant patients and 279 inactive status).

^c^Adjustments in multivariate models were made for age, sex, cardiac disease, hemiplegia, chronic pulmonary disease, connective tissue disorder, diabetes, mild liver disease, cirrhosis, HIV positive, peptic ulcer, any tumor without metastasis, hemodialysis at entry, central venous catheter, peripheral vascular disease, late referral, unemployed status, time on dialysis and listing periods (1984–1987 vs. 1988–1992 vs. 1993–1997 vs. 1998–2002 vs. 2003–2007 vs. 2008–2012).

^d^Adjusted for age and mild liver disease

^e^Inverse probability weighting estimates

## Discussion

This large cohort study of waitlisted candidates for KT demonstrates for the first time that patients waitlisted after a graft failure have an increased risk of mortality after listing compared with TN patients waitlisted for KT by using a competing risk approach and propensity score analyses. All the patients in our analysis were at some point on the KT waiting list, which makes the groups suitable for comparison. Accordingly, our findings will aid clinical decision making when considering relisted patients for another transplant.

The prevalence of patients waitlisted after graft failure (5.8%) was lower than that reported for this particular population, especially in other European and North American registries [2,4,5]. This lower prevalence rate could be due to the ample study period assessed in our cohort study (1987–2012), noting different clinical management in patients after graft failure over this long time. Whether high variability in practice patterns in country-specific dialysis and transplant recipient populations, as has been previously suggested [4,27], could justify these differences remains undetermined.

Although patients who return to dialysis after a failed graft have a higher risk of death while on the waiting list, the relationship between prior KT and mortality after relisting has not been explicitly assessed. Indeed, the comparison group in most previous reports was either KT recipients with a functioning graft or all patients undergoing dialysis, not just those on the waiting list, which could have attenuated the post-graft failure effect on mortality [4–6,8–11,13,28]. One large cohort study has assessed survival in patients waitlisted after graft failure, comparing them with primary KT candidates in a sensitivity analysis [5]. In that study, when the post-graft failure dialysis group was restricted to relisted patients, the mortality rate still remained significantly increased (32%) using conventional survival analyses. Similarly, another large study using conventional Cox regression methods found a higher risk of death in patients who return to dialysis after transplant failure [7]. However, neither of these studies undertook a competing risk analysis, as is warranted [15].

Although we adjusted for waitlist year in our multivariate competing risk analyses for mortality, we assessed an ample study period involving patients who were relisted between 1984–2012. Thus, they were likely managed with different clinical strategies during different transplant era, which could partly limit the generalizability of our findings. Indeed, when we performed a subanalysis of our study excluding those patients who were relisted between 1984–1988 and were under azathioprine-based immunosuppression, the adjusted competing risk regression model revealed a trend to statistical significance for mortality (SHR 1.42; 95% CI, 0.94–2.1; *P* = 0.09) in relisted patients after graft failure. This could reinforce the potential negative impact of a previous transplant on mortality, even in patients under CNI-based immunosuppression where a lower rejection rate was present, which provides more homogeneity to our study. At the same time, it could also suggest an improvement in the predialysis care of transplant patients with severe renal dysfunction in the more recent era. Accordingly, this could well explain why other authors have not observed a different mortality rate between relisted patients following graft failure compared with transplant-näive patients in more recent periods (2007–2009), as previously suggested [29]. Nevertheless, overall mortality in our relisted patients after graft failure was similar (16%) to the mortality rate observed in relisted patients (around 12%) during 2007–2009, as reported by Mourad et al in their contemporary cohort of patients [29].

In agreement with Rao et al [5], our patients waitlisted after graft failure had a 1.5-fold increased risk for all-cause mortality. Because these patients presented significant clinical differences compared with TN patients waitlisted for KT, we conducted propensity score analyses to limit the effect of selection bias by drawing similarly selected individuals from a large pool of TN patients waitlisted for KT. Indeed, two different marginal structural models, a propensity score-matched analysis and IPTW, were used. Matching has been shown to be the most effective way to use propensity scores for confounder adjustments. As expected, our matching strategy resulted in a virtually identical distribution of observed covariates. To reduce confounding that can occur with many to-one matching, we performed the analysis with 1:5 matching. We also matched patients closely by listing periods to eliminate immortal time bias [30]. We found a 1.6–fold increased risk of death among patients waitlisted after graft failure compared with matched TN waitlisted patients. As a sensitivity analysis, when the primary endpoint was modeled by IPTW using a competing risk approach, the risk of death for patients waitlisted after graft failure was almost identical. Nevertheless, despite the use of proper propensity score analyses for mitigating inclusion bias, we cannot completely exclude residual confounding when evaluating survival in observational studies.

We used a competing risk analysis, which may be more appropriate when assessing survival in waitlisted patients, especially in the presence of multiple competing events, as would be expected in KT candidates [15].

Although relisting after graft failure depends on different criteria worldwide, these patients have been reported to comprise a high-risk population group [7]. The majority of deaths in our relisted patients were due to cardiovascular disease, followed by infectious causes. This is consistent with reports suggesting that several risk factors, irrespective of traditional risk factors, could contribute to the persisting greater risk of death from cardiovascular and infectious causes [5,7,8,10]. Indeed, KT recipients are exposed to both uremia-related risk factors, such as elevated serum creatinine or proteinuria [31,32], and non-traditional risk factors inherent to transplantation, such as long-term immunosuppression [33]. Indeed, immunosuppression is a known risk factor for infection and malignancy and transplant failure patients are at very high risk for septicemia, mainly during the first 6 months after dialysis entry [9,10]. The fact that the risk for infection-related mortality is higher in relisted patients compared with transplant-näive waitlisted patients supports this argument [4]. In addition, suboptimal treatment of cardiovascular disorders after KT, related to under-utilization of angiotensin-converting-enzyme inhibitors and statins due to concerns of worsened allograft function could contribute to advanced arteriosclerosis and a higher mortality of relisted patients [7,34]. This stage could thus be a new clinical setting for a higher cardiovascular risk, where the cumulative exposure to these non-traditional risk factors may underlie the higher mortality in these patients. The fact that the patients relisted after graft failure were younger and had a lower baseline comorbidity burden at dialysis entry supports this view.

The presence of a failed renal allograft may be an ongoing source of low-chronic inflammation, an established risk factor for mortality in renal patients [35,36]. Additionally, an elevated risk of death is observed in patients who continue with low doses of immunosuppression after returning to dialysis in an attempt to preserve residual allograft renal function.

Interestingly, an increasing waiting-list time between first graft failure and second transplant has been associated with all-cause mortality post-transplantation [37]. A greater total time on dialysis was observed in our relisted patients compared with transplant-näive waitlisted patients. Additionally, a higher burden of immunosuppression in our cohort of relisted patients due to a higher glomerulonephritis rate could be expected. It is plausible, therefore, that both risk factors could have contributed to a higher mortality in our relisted patients. This, together with our results, suggests that reducing the waiting time between first graft failure and second or third KT could improve patient survival not only while on the list, but also post-transplantation. These results should be taken into account when assessing relisted patients for a second or third transplant, thereby possibly optimizing survival.

This study has other limitations. First, we did not record important risk factors for survival in patients undergoing dialysis who return to dialysis therapy after graft failure such as inflammatory markers (e.g. PCR levels), serum albumin, residual renal function at dialysis entry and panel reactive antibodies [1,8,11,38], or some community risk factors, such as obesity or smoking, which have been independently associated with mortality in the general population and waitlist patients [39]. Unfortunately, no data were available about transplant-related clinical information, such as immunosuppression, acute rejection or duration of transplant, which could have impacted on our results concerning mortality on the waiting list. Nor did we record cases developing new onset diabetes after transplantation, which confers a greater risk of death in patients on dialysis after graft failure [1,5]. However, we obtained comprehensive information about comorbidities included in the CCI and collected complete information on other clinical conditions and certain community risk factors. We included patients from a single region in southern Europe, almost exclusively white, which may limit the generalizability of our results.

## Conclusions

Patients on the waiting list for KT after graft failure have an increased risk of death compared with TN waitlist patients. This information will be useful in daily clinical practice when assessing relisted patients for a retransplant.

## Supporting information

S1 Table Clinical and demographic characteristics in patients who either received a kidney transplantation or were on the waitlist, including those with a previous graft failure and transplant-naïve waitlisted, at any time during follow-up. (DOCX)
