# Supplementary material for: Survival in Southern European patients waitlisted for kidney transplant after graft failure: A competing risk analysis
Source: PLoS One. 2018 Mar 7;13(3):e0193091. doi: 10.1371/journal.pone.0193091 (PMC5841738; doi:10.1371/journal.pone.0193091)
Supplement: S1 Table — (DOCX) [file pone.0193091.s001.docx]

**S1 Table**. Clinical and demographic characteristics in patients who either received a kidney transplantation or were on the waitlist, including those with a previous graft failure and transplant-naïve waitlisted, at any time during follow-up.

|  | **Waitlisted patients** (n=1876) | **KT patients** (n=1975) | ***P* value** |
| --- | --- | --- | --- |
| Mean age, *y* | 56.4±13 | 50±13 | <0.001 |
| Males, *%* | 62.5 | 63.6 | 0.492 |
| Cardiac disease*, *%* | 11.5 | 4 | <0.001 |
| Peripheral vascular disease, *%* | 11.4 | 4.8 | <0.001 |
| Hemiplegia, *%* | 3.4 | 0.8 | <0.001 |
| Chronic pulmonary disease, *%* | 8 | 2.4 | <0.001 |
| Connective tissue disorder, *%* | 6 | 2.4 | <0.001 |
| Diabetes | 21 | 13 | <0.001 |
| Mild liver disease^, *%* | 5 | 2 | <0.001 |
| HIV positive, *%* | 1.4 | 0.3 | <0.001 |
| Peptic ulcer, *%* | 3.5 | 2.2 | 0.012 |
| Any tumor without metastasis, *%* | 4 | 2 | <0.001 |
| Hemodialysis at entry, % | 83 | 77 | <0.001 |
| Central venous catheter, % | 42 | 27 | <0.001 |
| Previous transplant, % | 8.2 | 3.6 | <0.001 |
| Late referral**, *%* | 28 | 21.5 | 0.003 |
| Unemployed status^^, *%* | 65.5 | 50.5 | <0.001 |
| CCI score >3, *%* | 60 | 38.3 | <0.001 |
| Median total time on dialysis^+^ (IR), *mo* | 27 (12-48) | 20 (11-34) | <0.001 |

*Cardiac disease was considered as heart failure or myocardial infarction.

^Mild liver disease was considered as the presence of hepatitis B surface antigen or positive hepatitis C virus antibodies without cirrhosis.

** A 6-month cut-off time was defined for late referral to the nephrologist

^^Unemployed status was considered as unemployed or retired because of age or disability

^+^Total time on dialysis was defined as time from starting dialysis until end of follow-up, including time on waitlist for a first KT and time on dialysis whilst awaiting a second, third or fourth transplant. Thus, total time on dialysis represents the sum of all periods on dialysis throughout the follow-up.

Abbreviations: HIV, human immunodeficiency virus; CCI, Charlson comorbidity index; KT, kidney transplantation; IR, interquartile range
